# Evaluation of sonication for the detection of periprosthetic joint infection in hip revision arthroplasty: a retrospective observational single-center study of 288 episodes

**DOI:** 10.2340/17453674.2025.43679

**Published:** 2025-06-04

**Authors:** Bernhardt KICKINGER, Tor MONSEN, Emma KARIS, Micael WIDERSTRÖM, Petter SUNDIN, Kjell G NILSSON, Volker OTTEN, Sead CRNALIC

**Affiliations:** 1 Department of Diagnostics and Intervention, Umeå University; 2 Department of Clinical Microbiology, Umeå University, Sweden

## Abstract

**Background and purpose:**

Sonication fluid cultures have been proposed as a complementary diagnostic method to intraoperative tissue culture sampling for the diagnosis of periprosthetic joint infection (PJI). We evaluated whether sonication provides additional clinically relevant information in the diagnosis of PJI in hip revision.

**Methods:**

Episodes of hip revision performed between January 2007 and December 2016 were assigned retrospectively to the European Bone and Joint Infection Society (EBJIS) definition of periprosthetic joint infection: infection unlikely, infection likely, and infection confirmed. The inclusion criteria were a minimum of 2 perioperative tissue cultures collected at the index procedure and sonication performed on a removed implant. The results of the tissue cultures were compared with the results of the implant sonication fluid cultures (SFCs).

**Results:**

288 hip revision episodes in 250 patients fulfilled the inclusion criteria and were analyzed. The “infection unlikely” group included 203 episodes (178 patients), the “infection likely” group included 5 episodes (5 patients), and the “infection confirmed” group included 80 episodes (67 patients). SFC delivered additional clinical information in 15/288: 6 of 203 episodes in the “infection unlikely” group, 2 of 5 episodes in the “infection likely” group, and 7 of 80 in the “infection confirmed” group. Coagulase-negative staphylococci and *Staphylococcus aureus* were the dominant bacterial species in both the SFC and tissue cultures.

**Conclusion:**

In addition to tissue cultures, sonication fluid cultures optimized the microbiological yield in 15 out of 288 hip revision episodes.

Microbiologic diagnostics are a cornerstone of any postoperative therapy in periprosthetic joint infection (PJI). Despite the discovery of new markers such as alpha defensin and a refined new definition, including a validated scoring system for the diagnosis of PJI [1-4], the use of tissue cultures is regarded as gold standard in the diagnosis of PJI, and it has been shown that the tissue sample number increases sensitivity [5]. However, the overall low sensitivity of tissue cultures is recognized as a problem [6-8]. Several of the most common specimens isolated in PJI reside in biofilms that are built up around the implant [9].

Sonication is a complementary preparation step that dislodges bacteria and implant biofilm, thus enabling examination of the entire implant surface, whereas tissue cultures taken solely from the implant would assess only a limited surface area. Sonication fluid culture (SFC) has been shown to have a sensitivity of up to 94% in PJI diagnosis [10]. Most studies analyzed the performance of SFC in mixed materials from both knee and hip revisions [5,11,12]. The use of SFC should therefore not be interpreted as simply increasing the number of samples taken during the procedure. Another challenge in interpreting the value of SFC in hip revisions is different sonication protocols, different numbers of obtained tissue samples and different laboratory protocols used for tissue sampling and processing [5,6,8,13,14]. In addition, in studies that favor tissue sampling over sonication, the use of tissue sample yield improving automated liquid cultures has been described [5,11,12]. Since 2007, our department has performed sonication of explanted prosthesis parts according to the protocol presented by Monsen et al. [13,15].

The aim of our study was to evaluate the additional clinical information provided by sonication according to this protocol in hip revision.

## Methods

### Study design

We performed a retrospective single-center observational study evaluating the clinical value of sonication in the diagnosis of PJI in hip revision.

The guidelines of the STROBE statement were followed.

### Participants

We evaluated hip revision episodes performed at the Department of Orthopedics, Umeå University Hospital, Sweden, between January 2007 and December 2016. The indications for revision included suspected PJI, aseptic loosening, periprosthetic fracture, recurrent dislocations, and malignancy. In all episodes, simultaneous analysis of tissue cultures and SFC was performed. The inclusion criteria were hip revision performed at our department, at least 2 tissue samples taken, and sonication performed on a removed implant at the revision.

The included episodes were divided into 3 groups according to the 3-level approach suggested by the European Bone and Joint Infection Society (EBJIS) [16]: infection unlikely, infection likely, and infection confirmed. If a patient had more than 1 revision procedure performed during the observation period, each procedure was included as a separate episode and contributed to the analysis.

All patients’ charts were screened for meeting the criteria of “infection likely” or “infection confirmed” group according to the EBJIS classification. Episodes were assigned to each group with data available in the patients’ chart before and after the revision. If none of those criteria were met, the episode was assigned to the “infection unlikely” group.

The postoperative sonication results were not used to assign the episodes to either group.

The diagnosis of confirmed periprosthetic joint infection (PJI) in our study was based solely on clinical features and the microbiological criteria defined by the EBJIS classification. Other components of the EBJIS criteria—such as preoperative synovial fluid cultures, cytological analysis of synovial fluid, synovial biomarkers, nuclear imaging, and histological examination—were not routinely implemented in our department and therefore could not be applied.

The occurrence of potential re-revisions in the “infection unlikely” cohort subgroup was evaluated by examining the patient’s medical records in February 2021.

All episodes with < 3 months from the primary surgery to revision were described as early revisions; if the revision was performed between 3 and 24 months after primary surgery, the episodes were described as delayed revision; and revisions > 24 months after primary surgery were described as late revisions. Episodes described as debridement, antibiotics, and implant retention were classed as a hip revision as long as sonication was performed on a removed liner or head.

### Tissue sampling and sonication protocol

During the hip revision, a minimum of 2 standard tissue samples were taken from different locations in the hip joint, e.g., the synovia, the inside of the femur or underneath the cup. The sampling procedure was performed using new dedicated sterile instruments for each individual tissue sample (see detailed tissue sampling protocol and sonication protocol in Appendix).

### Microbiological characteristics

The outcomes of SFC were compared in respect of the total number of colony-forming units per mL (CFU/mL), and the bacterial species were identified. More than 200 CFU/mL was used as a cutoff value for sonicate fluid culture positivity, which is in accordance with the current literature [6,10]. The tissue cultures were compared by determining the presence or absence of bacteria/fungi, irrespective of the CFU counts.

### Added information when comparing tissue cultures and sonication

The outcome measure for the current study is the additional information that SFC provides in the diagnosis of PJI. To determine the additional information, all episodes were assigned to 1 of 2 groups.

#### Additional information obtained by implant sonication

The episodes included in group 1 were defined by meeting:

- Criterion 1: Bacterial specimens with > 200 CFU/mL were present in the SFC that were not detected in the tissue cultures, or
- Criterion 2: In the case of only 1 positive tissue culture for a bacterial specimen, at least 1 type of bacterial specimen found with > 200 CFU/mL in the SFC was identical to the specimen found in the tissue culture, thus supporting the tissue culture result and possibly ruling out contamination.

#### Additional information could not be obtained by performing implant sonication

Neither criterion 1 nor 2 is met.

### Statistics

This study was conducted as a retrospective observational analysis. Data was summarized using descriptive statistics, including measures of central tendency (e.g., mean, median). No inferential statistical analyses were performed.

### Ethics, funding, use of AI tools, and disclosures

This study was approved by the Regional Ethical Review Board at Umeå University (Dnr2013/122-31, 2015/48-31 and 2016-437-31 M). The study was funded by the County of Västerbotten and Medical Faculty at Umeå University. No conflict of interest is reported. Chat GPT 4o was used for rephrasing of solitary sentences. Complete disclosure of interest forms according to ICMJE are available on the article page, doi: 10.2340/17453674.2025.43679

**Figure 1:**
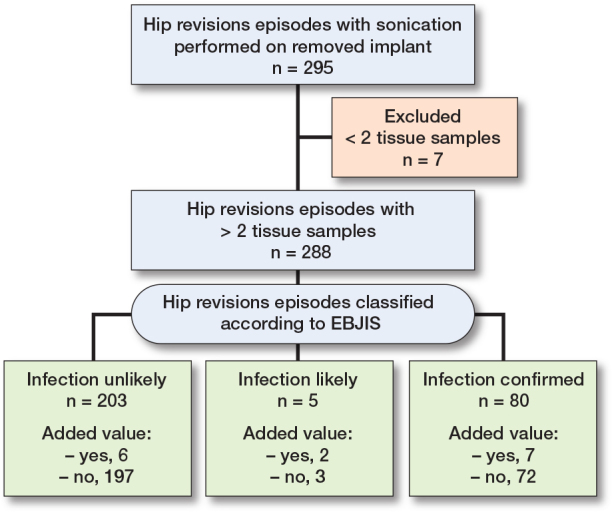
Patient flowchart.

## Results

The Figure describes the inclusion process and classification of patients undergoing hip revision surgery with sonication of removed implants. A total of 295 hip revision episodes were identified where sonication was performed on a removed implant. 7 episodes were excluded due to having fewer than 2 tissue samples, leaving 288 episodes (250 patients) for further evaluation. Episodes with 2 or more tissue samples (n = 288) were further classified based on the EBJIS criteria into 3 categories: infection unlikely (n = 203; 178 patients, median age 71 years), infection likely (n = 5; 5 patients, median age 78 years), infection confirmed (n = 80; 67 patients, median age 69 years). Each classification group was assessed for the added information.

The median follow-up time was 8.3 years (range 4.6–13.3) (Table 1).

**Table 1:** Patient demographics. Values are count (%) unless otherwise specified

Characteristics	Infection unlikely ^a^ (n = 203)	Infection likely ^a^ (n = 5)	Infection confirmed ^a^ (n = 80)
Median age (range)	69 (34–92)	78 (49–88)	72 (38–93)
Male/female sex, n	79/124	1/4	34/46
Diabetes mellitus	16 (7.9)	0	5 (6.3)
BMI, median (range)	26.3 (15-48)	28.8 (24–37)	27.3 (16–48)
ASA score, median	2	2	3
Presence of sinus tract	0 (0)	0	27 (34)
Exuding wounds for > 7 days	0 (0)	0	35 (44)
Preoperative antibiotics ^b^	5 (2.5)	2	27 (34)
Months of follow-up, median (range)	98 (56–59)	124 (64–159)	103 (57–158)
Months between primary surgery and revision, n			
0–3 (early)	7	1	37
4–24 (delayed)	29	2	19
>24 (late)	167	2	24

a According to EBJIS classification.

b < 2 weeks prior to revision.

ASA: American Society of Anesthesiologists; BMI: body mass index.

### Tissue sampling

The median number of tissue samples collected during surgery in all episodes was 6 (range 2–16). The number of tissue samples collected per case and episode with additional clinical information provided by sonication is described in brief in Tables 2 and 3.

**Table 2:** Microbiological findings in the “infection unlikely” group, laboratory assessment, additional treatment, and added information of sonication fluid culture

Episode	Tissue cultures ^a^	Laboratory assessment tissue	Sonicate-fluid culture ^b^	Laboratory assessment sonication	Additional treatment	Added information ^c^
1A	CoNS (1/6)	NS	Neg	Neg	Antibiotics and surgery	No
2A	*Neisseria* (1/2)	NS	Neg	Neg	No	No
3A	CoNS (1/8)	NS	Neg	Neg	No	No
4A	CoNS(1/6)	NS	Neg	Neg	No	No
5A	*Bacillus cereus* (1/4)	NS	Neg	Neg	No	No
6A	Micrococcus (1/7)	NS	Neg	Neg	No	No
	*Bacillus cereus* (1/7)					
7A	Micrococcus (1/7)	NS	Neg	Neg	No	No
8A	CoNS (1/2)	NS	Neg	Neg	No	No
9A	Neg (0/6)	Neg	C. acnes	S	No	Yes
10A	Neg (0/4)	Neg	C. acnes	S	No	Yes
11A	Neg (0/5)	NS	C. acnes	S	Antibiotics and surgery	Yes
12A	Neg (0/8)	Neg	Lactobacillus	S	Antibiotics	Yes
13A	Neg (0/2)	Neg	Micrococcus spp.	S	No	Yes
14A	Neg (0/6)	Neg	Microaerophilic streptococci	S	Antibiotics	Yes

C.: Cutibacterium; CFU: colony forming units; CoNS: Coagulase-negative staphylococci; Neg: Negative culture; NS: non-significant; S: significant.

a Number of positive tissue cultures/number of tissue cultures taken.

b Reported microbiological findings with cutoff 200 CFU/mL.

c Added clinical information due to sonication.

**Table 3:** Microbiological findings in “infection likely” and “infection confirmed” groups, laboratory assessment, additional treatment, and added information of sonication fluid culture

Episode	EBJIS classification: Infection	Prior antibiotics ^a^	Tissue cultures ^b^	Laboratory assessment tissue	Sonicate -fluid culture ^c^	Laboratory assessment sonication	Additional treatment	Added information ^d^
1B	likely	No	CoNS (1/6) Microaerophilic streptococci (1/6)	NS	*C. acnes*	S	Antibiotics	Yes
2B	likely	Yes	Neg (0/6)	Neg	*S. aureus*	S	Antibiotics and surgery	Yes
3B	confirmed	Yes	CoNS (7/14)	S	CoNS *Enterococcus faecalis*	S	Antibiotics and surgery	Yes
4B	confirmed	Yes	CoNS (1/7) –> *H. parainfluenzae* –>	NS S	CoNS *H. parainfluenzae*	S	Antibiotics	Yes
5B	confirmed	No	CoNS (16/16)	S	CoNS *S. aureus*	S	Antibiotic and surgery	Yes
6B	confirmed	No	Neg (0/9)	Neg	*C. acnes*	S	Antibiotics and surgery	Yes
7B	confirmed	No	Gram-negative rods (14/16)	S	CoNS	S	Antibiotics and surgery	Yes
8B	confirmed	No	Neg (0/6)	Neg	*Actinomyces spp.*	S	Antibiotics and surgery	Yes
9B	confirmed	No	Neg (0/9)	Neg	*C. acnes*	S	Antibiotics and surgery	Yes

For abbreviations, see Table 2 and S.: Staphylococcus

a Antimicrobial therapy within 14 days prior to index surgery.

b Number of positive tissue cultures/number of tissue cultures taken.

c Reported microbiological findings with cutoff 200 CFU/mL.

d Added clinical information due to sonication.

### Added information when using sonication fluid cultures

In the “infection unlikely” group, SFC generated additional information in 6 out of 203 episodes (Table 4). The median number of tissue samples per case in this group was 5; 2 cases had < 3 cultures taken.

**Table 4:** Added clinical information due to sonication fluid culture

Factor	Infection unlikely ^a^ (n = 203)	Infection likely ^b^ (n = 5)	Infection confirmed ^c^ (n = 80)
Criterion 1: specimen found			
in sonication (> 200 CFU/mL)			
but not in tissue cultures	6	2	5
Criterion 2: specimen found			
in only 1 tissue culture but			
the same specimen also found			
with sonication (> 200 CFU/mL)	0	0	2
Total number of episodes with			
added clinical information			
provided by sonication	6	2	7

CFU/mL: colony forming units per milliliter of sonicate fluid.

a See Table 2, Cases 9A–14A.

b See Table 3, Cases 1B–2B.

c See Table 3, Cases 3B–9B.

In the “infection likely” group, SFC generated additional information in 2 of the 5 episodes. Both cases had 5 tissue samples taken.

In the “infection confirmed” group, SFC generated additional information in 7 of the 80 episodes. The median number of tissue samples per case in this group was 9. No case had < 3 cultures taken.

7 cases in the “infection likely” and the “infection confirmed” group had negative SFC at the same time.

In early revisions, SFC-generated additional information was found in 1 of 45 episodes, in delayed revisions additional information was found in 5 out of 50 episodes, and in the late revisions additional information was found in 9 out of 193 episodes.

### Bacterial microorganisms that could be isolated in either sonication fluid or tissue cultures

The 3 dominant pathogens were coagulase-negative staphylococci (CoNS), *Staphylococcus aureus* and *Cutibacterium acnes* (Tables 5 and 6). *C. acnes* was isolated in the SFC from 9 episodes but only in the tissue cultures from 3 episodes. *Lactobacillus spp.* was only found in SFC, while *Streptococcus agalactiae* (group B streptococcus), *Streptococcus salivarius,* and *Klebsiella pneumonia*e were isolated only in the tissue cultures. Negative cultures were observed in the sonication fluid from 212 episodes, and negative tissue cultures were observed in 221 episodes.

**Table 5:** Microbiological findings in sonication fluid culture from 288 hip revisions in relation to EBJIS classification groups and whether preoperative antimicrobial treatment was prescribed within 14 days prior to surgery

Specimen	Sonication fluid culture Infection				Preoperative antibiotics ^b^	
	n	confirmed ^a^	likely ^a^	unlikely ^a^	No	Yes
CoNS	40	28	2	10	31	9
*Staphylococcus aureus*	20	19	1	0	10	10
*Cutibacterium acnes*	9	5	1	3	9	0
*Enterococcus faecalis*	6	6	0	0	5	1
*Streptococcus mitis*	3	2	0	1	3	0
*Corynebacterium spp*.	2	2	0	0	1	1
*Peptostreptococcus sp*p.	2	2	0	0	2	0
*Micrococcus spp*.	2	0	0	2	2	0
Alfa hemolytic streptococci	2	1	0	1	2	0
*Bacillus cereus*	1	1	0	0	1	0
*Bacteroides fragilis*	1	1	0	0	1	0
*Enterobacter cloacae*	1	1	0	0	1	0
*Escherichia coli*	1	1	0	0	1	0
*Actinomyces spp.*	1	1	0	0	0	1
Streptococci Group G	1	1	0	0	0	1
*Hemophilus parainfluenzae*	1	1	0	0	0	1
*Pasteurella multocida*	1	1	0	0	0	1
*Streptococcus anginosus*	1	1	0	0	1	0
*Serratia marcescens*	1	1	0	0	1	0
*Proteus mirabilis*	1	0	1	0	0	1
Microaerophilic streptococci	1	0	0	1	1	0
*Lactobacillus sp*p.	1	0	0	1	1	0
Gram negative rods	1	0	0	1	1	0
Culture negative	212	19	1	192	197	15

CoNS: Coagulase-negative staphylococci.

a EBJIS classification groups.

b Patient received antibiotic treatment up to 14 days prior to the revision.

**Table 6:** Microbiological findings in tissue culture from 288 hip revisions in relation to EBJIS classification groups and whether preoperative antimicrobial treatment was prescribed within 14 days prior to surgery

Specimen	Tissue culture Infection				Preoperative antibiotics ^b^	
	n	confirmed ^a^	likely ^a^	unlikely ^a^	No	Yes
CoNS	42	33	3	6	34	8
*Staphylococcus aureus*	20	20	0	0	10	10
*Enterococcus faecalis*	7	7	0	0	5	2
*Bacillus cereus*	5	3	0	2	3	2
*Cutibacterium acnes*	3	3	0	0	3	0
*Micrococcus spp.*	3	3	0	0	3	0
*Streptococcus mitis*	3	3	0	0	3	0
*Corynebacterium spp*.	2	2	0	0	1	1
*Peptostreptococcus spp.*	1	1	0	0	1	0
Alfa hemolytic streptococci	1	1	0	0	1	0
*Bacteroides fragilis*	1	1	0	0	1	0
*Enterobacter cloacae*	1	1	0	0	1	0
*Escherichia coli*	1	1	0	0	1	0
Gram negative rods	1	1	0	0	1	0
Streptococci Group G	1	1	0	0	0	1
*Hemophilus parainfluenzae*	1	1	0	0	0	1
*Pasteurella multocida*	1	1	0	0	0	1
*Serratia marcescens*	1	1	0	0	1	0
*Streptococcus anginosus*	1	1	0	0	1	0
*Streptococcus salivarius*	1	1	0	0	1	0
*Klebsiella pneumoniae*	1	1	0	0	0	1
*Proteus mirabilis*	1	0	1	0	0	1
Microaerophilic streptococci	1	0	1	0	1	0
Streptococci Group B	1	0	0	1	1	0
*Neisseria spp.*	1	0	0	1	1	0
Culture negative	221	19	2	200	205	16

See footnote for Table 5.

### Isolated microorganisms and re-revision in the “infection unlikely” group

In 8 out of 203 episodes, bacteria were isolated in the tissue cultures, while the SFCs were negative (Table 2). These episodes were classed as “infection unlikely” as no other criteria according to the EBJIS classification group “infection likely” were met besides 1 single positive culture. In 1 episode, CoNS was isolated in the tissue cultures only. This episode was later treated with DAIR due to wound leakage and suspected infection (follow-up: 6.1 years). In the other 7 episodes, the tissue culture results were considered to be putative contamination, and no treatment was initiated postoperatively.

In 6 out of 203 episodes, SFCs were positive (> 200 CFU/mL) and thus interpreted by the laboratory as significant, but the tissue cultures were negative (Table 2). In 2 of those episodes (Table 2), antibiotic treatment was initiated due to the sonication findings of *Lactobacillus ssp.* and Microaerophilic streptococci, respectively. No further surgery was performed in those 2 episodes (follow up time of 12.9 and 10.8 years). In 2 episodes where the SFCs showed significant growth of *C. acnes,* no antimicrobial therapy was initiated (Table 2) and no further surgery was performed. 1 episode with unexpected growth of *C. acnes* in the SFC alone received antibiotic treatment; this patient underwent re-revision 7 years later due to an unrelated periprosthetic fracture (Table 2). All implants were still functional with a median follow-up of 9.8 years (range 4.9–12.9).

### Evaluation of the value of sonication fluid culture in the “infection confirmed” and “infection likely” groups

In 2 episodes in the “infection likely” group and 7 episodes in the “infection confirmed” group (Table 3), SFC provided additional microbiological information compared with tissue cultures. In 4 of these episodes, the tissue culture results were negative (Table 3). In 5 episodes with significant findings in the tissue cultures, SFC increased the detection of polymicrobial infection either by amplifying nonsignificant findings in the tissue cultures (1 case, Table 3) or by detecting additional bacterial species (4 cases, Table 3).

## Discussion

To our knowledge, this is one of the largest studies to date investigating sonication results in hip revision only. The aim of the present study was to evaluate whether sonication of implants could provide additional clinical information in the diagnosis of PJI in hip revision surgery. We found that SFC provided information in 9 out of 85 hip revisions classified as “infection likely” or “infection confirmed” but not to the same extent in hip revisions classified as “infection unlikely” (6 out of 203) according to the EBJIS classification. The use of sonication was helpful for evaluating contamination in 8 episodes in the “infection unlikely” group. In the case of only 1 positive tissue culture in these episodes, the absence of these findings in the SFC strengthens our belief of suspected tissue culture contamination.

Overall, SFC added clinical information in 5% of the episodes (15/288 episodes, 250 patients), which is in line with other studies [14]. We found that in 9 of 85 episodes with hip revision classified as “infection likely” or “infection confirmed,” sonication provided additional clinically useful information compared with tissue cultures only. These findings show that SFC may provide more information in suspected and confirmed PJI than in cases where infection is unlikely according to the EBJIS classification.

Our results show that the standardized use of SCF as a diagnostic tool is of limited value in hip revision classified as “infection unlikely,” as it provided additional information in only 6 of 203 (3%) episodes. The limited clinical value gained by SCF in this group and the promising results in the follow-up of these patients may suggest that sonication is not cost-effective in this patient cohort. In particular, episodes initially classified as “infection unlikely” with findings of *C. acnes* and *Micrococcus spp.* in SFC and negative tissue cultures had uneventful follow-up without antimicrobial treatment. Despite the used cut-off value of 200 CFU/mL in the sonication fluid, contamination in these samples must be suspected. These findings correspond with other reports suspecting the prolonged exposure of implants to the air of the operating room during implant removal as possible cause of contamination [17]. The overall clinical significance of *C. acnes* exclusively in SFC remains to be elucidated [18].

Only a few studies have described microbiological yield in hip revision through sonication in a large patient cohort [19,20]. In the present study, the most common pathogen found in both tissue cultures and sonication was CoNS, followed by *S. aureus,* and these results are in accordance with previous publications [21,22]. Our results show that sonication of hip implants seems to be more sensitive for the detection of *C. acnes* than tissue culture sampling. *C. acnes* is known to cause low virulent implant infections through a biofilm mode of growth [23], and biofilms may be mechanically disrupted by sonication [24].

2 episodes with uncommon microbiological findings were identified in the PJI group: coinfection with *Haemophilus parainfluenzae* and CoNS, and PJI due to *H. parainfluenzae,* which is part of the normal flora in the human upper airway, has rarely been reported [25]. 1 episode of coinfection with *Pasteurella multocida* and CoNS was identified although in this episode SFC provided no additional value as both pathogens were detected by at least 2 positive tissue cultures.

### Strengths

Several other studies comparing sonication vs tissue cultures applied different patient inclusion criteria [5,10,19,26]. These criteria have varied from acute and chronic PJI in the knee, hip, and shoulder to chronic osteomyelitis and infected fractures, thus making it difficult to apply the results to hip revision. All patients underwent the procedure at the same unit, and all sonication procedures were performed at the same department of microbiology. The well-documented follow-up as well as the long follow-up time of all patients makes the outcome analysis reliable.

### Limitations

At the start of the study, preoperative synovial fluid cultures, synovial fluid cytological analysis, synovial biomarkers, nuclear imaging, and histology, described in the EBJIS definition of prosthetic joint infection, were not in standard use at our department [16]. It was therefore not possible to directly calculate the sensitivity and specificity of sonication vs tissue cultures in relation to available preoperative data.

### Conclusion

In addition to tissue cultures, sonication fluid cultures optimized the microbiological yield in 15 out of 288 cases of suspected and confirmed PJI.

*In perspective,* sonication may optimize the diagnosis of PJI but tissue cultures should remain the gold standard.

## Notes

BK, TM, MW, KGN, VO, and SC conceived and designed the study and analyzed and interpreted the data. EK and PS collected the clinical data. BK and SC drafted the manuscript. TM and MW were responsible for the initial reviewing and editing. All authors read, revised, and approved the manuscript before submission.

Handling co-editors: Marianne Westberg and Robin Christensen

*Acta* thanks Mats Bue and Martin McNally for help with peer review of this manuscript.

## Appendix

### Tissue sampling

The biopsies were placed in glass tubes with fastidious anaerobic broth (Neugen, London, UK) and were transported to the local clinical microbiological laboratory.

On arrival, 100 μL of the anaerobic transport media was distributed on McLeod/hematin-agar (Difco, Atlanta, GA, USA) and was incubated until visible growth or for a maximum of 48 hours in 5% CO_2_ at 37°C. The anaerobic transport tubes were incubated further for enrichment at 37°C for 48 hours under anaerobic conditions with a slightly open tube capsule. Then, 1 μL of anaerobic transport medium was spread on pre-reduced fastidious anaerobic agar (Neugen, UK) supplemented with 5% horse blood, and the agar was incubated for 96 hours/4 days at 37°C under anaerobic conditions. The aerobic blood agar (Columbia blood agar base, BD, USA) was supplemented with 5% horse blood and was incubated at 37°C in air for 24 hours. Visible colonies were picked for further examination.

### Sonication protocol

The explanted metal and plastic orthopedic components were collected in a sterile bag or container filled with 5 mL of sterile saline to avoid drying of the explant and were transported to the laboratory. The implants were then transferred to glass sonication tubes filled with sterilized sonication phosphate buffer until the implant was covered. All containers were sealed to minimize contamination risk. The sonication tubes were then submerged in a larger, water-filled sonication tank with an ultrasound generator at the bottom. The sonication procedure was performed according to the protocol by Monsen et al. [13]. Briefly, sonication was performed at 40 kHz at 350 W for 7 minutes. The sonication fluid was repeatedly centrifuged for 15 minutes in 50 mL plastic tubes (concentrated). The final pellet was resuspended in 400 μL sterilized saline; 100 μL each was distributed to blood, hematin/chocolate, and anaerobic agar plates. The plates were incubated under aerobic (5% CO_2_) and anaerobic conditions for 7 days. Bacterial identification was performed in accordance with standard laboratory practice using matrix-assisted laser resorption/ionization time-of-flight mass spectrometry (MALDI-TOF MS) with a Microflex LT (Bruker Daltonik GmbH, Bremen, Germany) and MALDI Biotyper software v3.1 DB7311 (Bruker Daltonik) according to the manufacturer’s instructions. A score > 2.0 was required for species identification. The number of CFU on the plate with most positive findings/most colonies (CFU) in 100 μL was then multiplied by 10 (= concentration/mL of concentrated sonication fluid). The outcome of SFC is presented as the quantities of colony forming units per mL of concentrated sonicate fluid.
